# Magneto-optical diagnosis of symptomatic malaria in Papua New Guinea

**DOI:** 10.1038/s41467-021-21110-w

**Published:** 2021-02-12

**Authors:** L. Arndt, T. Koleala, Á. Orbán, C. Ibam, E. Lufele, L. Timinao, L. Lorry, Á. Butykai, P. Kaman, A. P. Molnár, S. Krohns, E. Nate, I. Kucsera, E. Orosz, B. Moore, L. J. Robinson, M. Laman, I. Kézsmárki, S. Karl

**Affiliations:** 1 Institute of Natural Materials Technology, University of Technology, Dresden, Germany; 2 Vector-borne Diseases Unit, PNG Institute of Medical Research, Madang, Madang Province Papua New Guinea; 3 Department of Physics, Budapest University of Technology and Economics, Budapest, Hungary; 4 Australian Institute of Tropical Health and Medicine, James Cook University, Smithfield, QLD Australia; 5 Experimental Physics 5, Center for Electronic Correlations and Magnetism, University of Augsburg, Augsburg, Germany; 6 National Public Health Center, Budapest, Hungary; 7 School of Pharmacy, Curtin University, Bentley, WA Australia; 8 Burnet Institute, Melbourne, VIC Australia

**Keywords:** Biophysical methods, Infectious-disease diagnostics, Parasitology, Malaria, Imaging and sensing

## Abstract

Improved methods for malaria diagnosis are urgently needed. Here, we evaluate a novel method named rotating-crystal magneto-optical detection (RMOD) in 956 suspected malaria patients in Papua New Guinea. RMOD tests can be conducted within minutes and at low cost. We systematically evaluate the capability of RMOD to detect infections by directly comparing it with expert light microscopy, rapid diagnostic tests and polymerase chain reaction on capillary blood samples. We show that compared to light microscopy, RMOD exhibits 82% sensitivity and 84% specificity to detect any malaria infection and 87% sensitivity and 88% specificity to detect *Plasmodium vivax*. This indicates that RMOD could be useful in *P. vivax* dominated elimination settings. Parasite density correlates well with the quantitative magneto-optical signal. Importantly, residual hemozoin present in malaria-negative patients is also detectable by RMOD, indicating its ability to detect previous infections. This could be exploited to reveal transmission hotspots in low-transmission settings.

## Abstract (web summary)

Here Arndt et al. establish rotating-crystal magneto-optical detection (RMOD) as a near-point-of-care diagnostic tool for malaria detection and report a sensitivity and specificity of 82% and 84%, respectively, as validated by analyzing a clinical population in a high transmission setting in Papua New Guinea.

## Introduction

Humans have suffered from malaria for thousands of years and still, hundreds of millions of people are infected each year. Nowadays, malaria also places a significant social and economic burden on many tropical developing countries, further undermining the potential for growth^1^. Development of rapid, easy-to-use and low-cost malaria diagnostic methods, with high sensitivity and specificity, remains an urgent priority in tropical diseases research^2,3^. Currently available methods include the inspection of blood smears using light microscopy (LM), rapid diagnostic tests (RDTs), and molecular methods, such as polymerase chain reaction (PCR) or other molecular techniques. These techniques rely on different diagnostic targets, namely the direct observation of infected red blood cells (LM), detection of parasite antigens (RDT), or DNA/RNA (using PCR or other molecular techniques).

Researchers have also been fascinated by the magnetic properties of malaria-infected red blood cells (RBCs) for a long time, since using an inherent and unique physical property such as malaria parasite-induced RBC magnetism, may enable rapid and easy diagnosis at low cost. The increased magnetic susceptibility of RBCs infected with *Plasmodium* parasites is a striking and well-described biophysical phenomenon, arising from the metabolism of hemoglobin^4–6^. Normally, oxygen-bound hemoglobin is a diamagnetic substance with a magnetic susceptibility close to that of water^7^. During infection of RBCs, *Plasmodium* parasites break down hemoglobin in their digestive vacuoles and heme molecules liberated in the process are assembled into iron-containing organic crystallites called hemozoin^2,8–11^. These hemozoin crystals are one of the most distinguishing features of *Plasmodium* infection in peripheral blood and played a vital role in identifying *Plasmodium* parasites as the cause of malaria and the mosquito as the agent of transmission in the late 19th century^12,13^. During the process of hemozoin formation, hemoglobin iron is oxidized and concentrated to make up about 10% of the mass of the newly formed hemozoin crystals, resulting in an overall paramagnetic behavior of hemozoin^4^. As such, hemozoin is an intrinsic biomagnetic marker of infection with *Plasmodium*. While the hemozoin crystals are not excreted naturally by the infected cells until cell rupture, they can easily be made accessible for diagnostic purposes by lysing blood.

Several approaches to exploit these magnetic properties for diagnostic purposes have been proposed^14–28^ and some of these techniques showed promise under laboratory conditions. However, only a few magneto-optical (MO) methods have been tested in operational settings in malaria endemic countries or with samples collected from malaria-infected patients in endemic countries. A promising concept for hemozoin-based malaria diagnosis was described by Newman and colleagues in 2008^18^. This applicability of the method was demonstrated using synthetic hemozoin (β-hematin) samples, but the ability to detect infections in a set of LM-confirmed patient samples under laboratory conditions, was considered requiring further development^16^. Building upon this concept, over recent years, we developed a novel diagnostic technique, named rotating-crystal magneto-optical detection (RMOD). As detailed in refs. ^23,25,26^, the main conceptual improvements were (i) synchronous magnetic rotation of the hemozoin crystals using an array of permanent magnets, (ii) application of a circularly polarized incoming laser beam to maximize the MO signal and to avoid contributions from components other than hemozoin, and (iii) highly efficient detection of the MO signal originating from hemozoin by a balanced photodetection scheme combined with lock-in filtering. With these improvements, we showed that RMOD can detect hemozoin in very small concentrations of a few ppm. Furthermore, we demonstrated that hemozoin can be detected in lysed blood with very high sensitivity in the low ng/µL range using RMOD^26^. Measurements on parasite cultures indicated that RMOD had a limit of detection (LOD) of ∼10 parasites per µL of blood in samples spiked with *P. falciparum*^26^. These promising results were further supported by studies on *P. berghei* and *P. yoelii*-infected mice^23,24^. On an operational level, RMOD is promising as it can be conducted after a short training and provide test results within minutes. From a funding perspective, since no expensive reagents are used, the per-sample measurement cost is very low.

The dynamics of hemozoin accumulation and clearance in natural malaria infections in the human body are more complex than what can be mimicked in model systems, and intermediate redistribution and final fate of hemozoin during and following malaria infections are not well-understood^29^. Many mechanisms and intricacies of parasite biology and human response to infection may collectively determine the actual quantity of hemozoin in peripheral blood. For example, infected RBCs are cleared mostly in the liver and spleen, and consequently these organs become loaded with hemozoin^30,31^. Leukocytes phagocytose infected RBCs and ingest hemozoin^32,33^. Late parasite stages of *P. falciparum* containing large quantities of hemozoin sequester in the capillaries^34^, whereas a higher proportion of late stages of *P. vivax* continues to circulate in peripheral blood. Gametocytes of *P. falciparum* contain large amounts of hemozoin and circulate for an extended period of time after an infection has been cleared or treated^35,36^. While many aspects of hemozoin clearance and redistribution in the human body are yet to be elucidated, there is evidence for long-term persistence of hemozoin in body tissues of people living in endemic areas^31,37^.

In order to account for these complexities, which are potentially relevant to hemozoin-based malaria diagnosis, we conducted a detailed evaluation of RMOD on almost 1000 suspected malaria cases in Madang, Papua New Guinea (PNG). PNG has a complex malaria epidemiology and the study area exhibits high transmission intensities for both, *P. falciparum* and *P. vivax*^38^. Using a RMOD prototype device similar to that described in our previous studies^23–26^, we systematically compared RMOD performance to conventional diagnostic techniques, namely expert LM, RDT, and PCR.

**Fig. 1:**
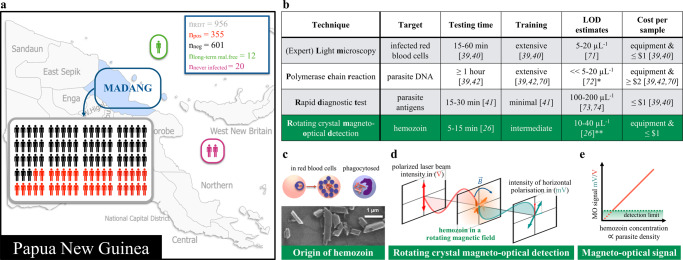
Overview of the present study. **a** Comparison of existing malaria diagnostic techniques to RMOD (**b**), and working principle of RMOD (**c**–**e**).** a** Overview of the study population in Madang, Papua New Guinea (base map was created with paintmaps.com). A total of 956 suspected malaria cases were enrolled of whom 355 were found positive by RDT. Samples from 32 malaria naïve and long-term malaria-free patients were also included. **b** Comparison of existing diagnostic techniques with RMOD in terms of their target, approximate testing time^26,39–42^, level of training^39–42,70^, limit of detection^26,71–74^, and cost per sample^39–42,70^. * LOD of PCR depends on the volume of blood subjected to the reaction and can be much lower; ** LOD determined using dilution series of *Plasmodium falciparum* cultures. **c** Hemozoin crystals formed by *Plasmodium falciparum*, as imaged by scanning electron microscopy. The scanning electron microscopy image was produced by the authors based on the methodology described in Orban et al.^26^ with slight modifications. In peripheral blood, hemozoin can be present inside infected red blood cells or phagocytosed in leukocytes. **d** RMOD principle. Polarization of the incoming laser beam is tilted due to the linear dichroism of hemozoin crystals. The rotating magnet drives a synchronous rotation of the crystals in lysed blood, which leads to a periodic tilting of the polarization resulting in a periodic modulation of the intensity of the outgoing laser beam. **e** The ratio of the modulated intensity and the mean intensity provides the magneto-optical (MO) signal in mV/V, which is a highly sensitive quantitative measure of hemozoin concentration and, thus, proportional to the parasite density.

Figure 1 provides an overview of the study site and population (Fig. 1a); a brief comparison of conventional diagnostic techniques together with RMOD (Fig. 1b)^4,26,39–42^, and a schematic illustration of the RMOD measurement principle (Fig. 1c–e).

In this study, we establish that magneto-optical hemozoin detection using RMOD is a promising approach for clinical and in-field malaria diagnosis, exhibiting a sensitivity and specificity of 82% and 84%, respectively.

## Results

### Study population and infection data

A total of 956 suspected malaria patients were enrolled into the study. All of them had an RDT result, while 945 had a LM result and a PCR result. The overall properties of the patient population, and the malaria diagnostic and parasitological results are given in Table 1.

**Table 1:** Characteristics of the study population and infection data (*n* = 956).

*Study population characteristics (n = 956)*^a^		
Age (years, median, range)	**21** (2–80)	
Weight (kg, median, range)	**46** (9–100)	
Hemoglobin^b^ (g dL^−1^,median, range)	**9.3** (2.8–16.4)	
Female (%, n)	**59.3** (567)	
Fever (% with >37.5 °C, *n*)	**16.7** (159)	
Anemia^b^ (% with Hb < 11.0 g dL^−1^, *n*)	**81.4** (544)	
Recent malaria^c^ (% proportion, *n*)	**44.9** (429)	
*Rapid diagnostic test (n* *=* *956) % positive (n)*		
RDT positive	**37.1** (355)	
HRP2	**6.3** (60)	
pLDH	**10.3** (98)	
HRP2 + pLDH	**20.6** (197)	
*Light Microscopy (n* *=* *945)*	***% positive*** *(n)*	***Parasite density*** *(µL*^−1^, geometric mean, range)
Any infection	**33.7** (318)	**4005** (16–111,300)
*P. falciparum*	**20.4** (193)	**5957** (16–111,300)
*P. vivax*	**11.0** (104)	**1637** (16–36,420)
*P. falciparum* + *P.vivax*	**2.1** (20)	**9818** (1015–52,349)
*P. malariae*	**0.1** (1)	**4800** (–)
*P. f. gametocytes*	**6.7** (63)	**276** (16–6520)
*P. v. gametocytes*	**5.9** (56)	**168 (**32–1349)
*PCR (n* *=* *945)*	***% positive*** *(n)*	***18S rRNA*** ***copy number***^d^ (µL^−1^, geometric mean, range)
Any infection	**34.3** (324)	**3260** (0.5–3.3 × 10^6^)
*P. falciparum*	**20.8** (197)	**9196** (4-3.3 × 10^6^)
*P. vivax*	**10.8** (102)	**809** (0.5–1.7 × 10^5^)
*P. falciparum* + *P.vivax*	**2.6** (25)	**2797/316** (2–1.3 × 10^5^/2–3.5 × 10^4^)

^a^Bold numbers stand for percentages or medians, while numbers in parentheses indicate ranges or number of patients

^b^Hb/Anemia could only be determined for *n* = 674 patients

^c^Based on patient self-reporting

^d^The copy number of the *18S rRNA* gene target employed in this study is up to 5 per genome.

Overall, based on LM diagnosis 34% of patients were positive for any malaria infection, as compared to 37% and 34% by RDT and PCR methods, respectively.

Among the three reference methods used in this study, RDT was applied at enrollment and expert LM was used as the main reference method, which is the gold standard recommended by WHO^42^.

### Comparison of conventional diagnostic methods

When LM was used as the reference standard for comparison with RDT and PCR methods, RDT exhibited a sensitivity of 87% and specificity of 88%, while PCR showed a sensitivity of 80% and specificity of 89%. A table showing the commonly used measures of agreement (sensitivity, specificity, predictive values, and Cohen’s κ) is included as Supplementary Table 1.

For the quantitative methods, LM and PCR, parasite density and gene copy number correlated well (Spearman Rank Correlation: *P. falciparum* R2 = 0.79; *p* < 0.0001; *P. vivax:* R2 = 0.71; *p* < 0.0001). A correlation plot is shown in Supplementary Fig. 1.

### RMOD results

**Fig. 2:**
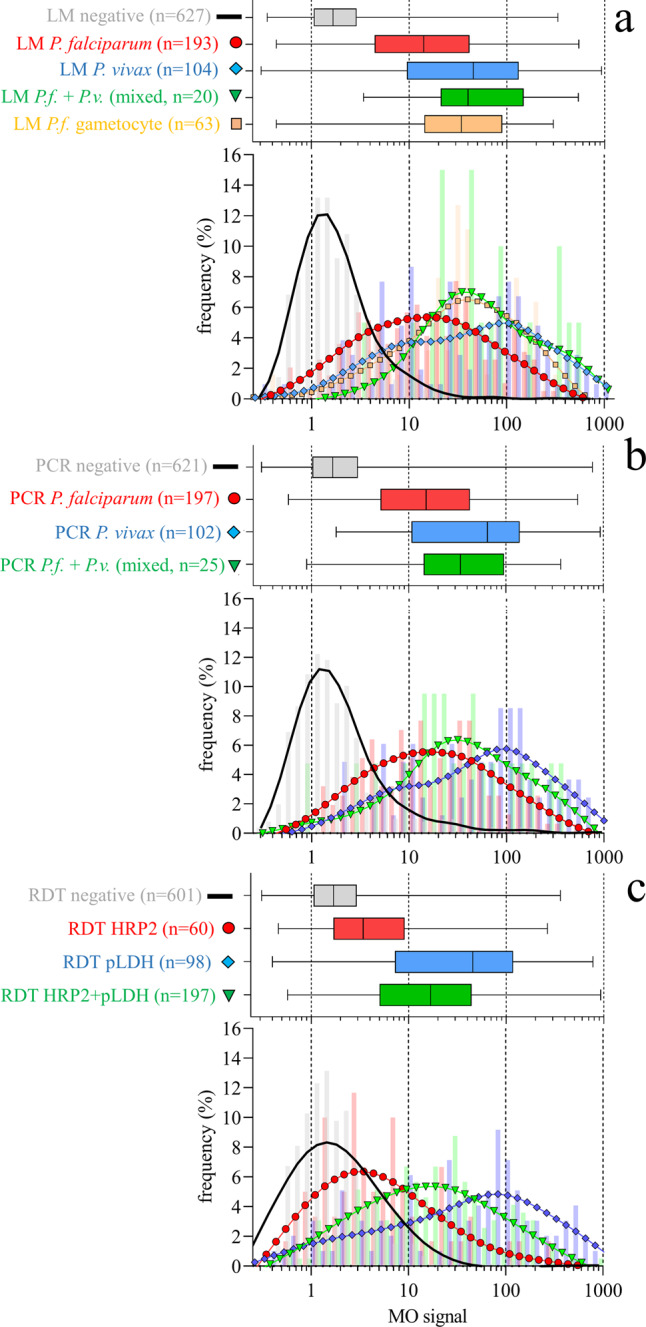
RMOD data in comparison to different reference methods. **a** Expert LM, (**b**) PCR and (**c**) RDT. The panels are divided into box-and-whisker plots (top) and histograms (bottom). The box-and-whisker plots show median, interquartile range (IQR), and range of the MO signals for the respective diagnostic results. The histograms show the raw distribution of MO signal data (bars) and a smoothened line resulting from applying generalized additive models of the MO signal frequency distributions for the respective diagnostic results. All panels share a common horizontal scale of MO signal. Color and symbol coding for each panel is given by the font color and the symbols next to the labels of the box-and-whisker plots. The number of independent biological samples is given as part of each label. Each biological sample was measured in triplicate.

The median overall magneto-optical signals for LM positive and LM negative samples were 23.0 mV/V and 1.7 mV/V, respectively. For the RDT positive and negative samples the median MO signals were 15.1 mV/V and 1.7 mV/V, respectively. For PCR positive and negative samples the median MO signals were 16.4 mV/V and 1.7 mV/V, respectively. For all three methods, the differences in the median MO signals for positive and negative samples were highly statistically significant (Mann–Whitney test *p-*values < 0.0001). A detailed summary of the RMOD results compared to the other methods (LM, RDT, PCR) is shown in Fig. 2.

We note that the typical error of the RMOD test for a given sample (based on measurement of three individually prepared replicates per sample) is two orders of magnitude smaller than the dynamic range of the methodology defined as the difference between the median MO signal of the LM negative and LM positive population. The distribution of the standard deviations of the MO signals, as determined from measurements of triplicates, is presented for the whole sample set in Supplementary Fig. 3.

Since RMOD provides a quantitative measurement of hemozoin content in the blood sample and the detected MO signals are continuous, receiver operating characteristic (ROC) analyses were conducted to determine the cut-off MO signals corresponding to maximum sensitivity and specificity to detect infection per se, as well as infection with specific parasite species.

Sensitivity and specificity were determined by selecting the minimum distance from the ROC curve to the [0,1] coordinate indicating 100% sensitivity and specificity. The cut-off values and resulting indicators of agreement between methods are presented in Table 2. (A list of the predictive values and the area under the ROC curve is provided in Supplementary Table 2). The ROC curves are included as Supplementary Fig. 2.

**Table 2:** Cut-off values, and estimated sensitivity and specificity of RMOD resulting from ROC analysis performed using different reference methods (LM, PCR, RDT) and different infection characteristics.

	Cut-off (mV/V)^a^	Sensitivity (%)^a^	Specificity (%)^a^	κ^a,b^
*LM result*				
all LM positive	4.19	**82** (78–86)	**84** (81–86)	**0.64**
*P. falciparum*	4.00	**79** (73–84)	**83** (80–86)	**0.57**
*P. vivax*	5.15	**87** (80–92)	**88** (85–90)	**0.63**
*P.f* + *P.v*. (mixed)	10.20	**95** (76–100)	**96** (94–97)	**0.55**
*PCR result*				
all PCR positive	3.54	**78** (73–82)	**78** (75–81)	**0.54**
*P. falciparum*	3.01	**80** (74–85)	**75** (71–78)	**0.47**
*P. vivax*	4.84	**79** (71–86)	**84** (81–87)	**0.51**
*P.f.+P.v*. (mixed)	5.40	**80** (61–91)	**85** (82–88)	**0.24**
*RDT result*				
all RDT positive	4.11	**76** (71–80)	**83** (79–86)	**0.58**
PfHRP2	2.58	**65** (52–77)	**70** (66–73)	**0.16**
pLDH	5.14	**81** (71–88)	**87** (84–90)	**0.55**
PfHRP2+pLDH	4.11	**81** (75–86)	**83** (79–86)	**0.57**

^a^Bold numbers are the means and numbers in parentheses are the 95% confidence intervals

^b^κ is the coefficient of agreement according to Landis and Koch^43^.

In summary, when based on expert LM as reference standard, ROC analysis indicated that maximum sensitivity and specificity of RMOD for the detection of any *Plasmodium* infection were 82% and 84%, respectively, and the optimal cut-off value to distinguish between infections and non-infections was 4.19 mV/V. The overall agreement between LM and MO was classified as substantial (κ = 0.64)^43^. Sensitivity and specificity increased for *P. vivax* infections (incl. mixed infections) to 87% and 88%, respectively. When based on PCR as a reference method, overall sensitivity was 78% and overall specificity was 78%. The overall agreement between PCR and MO was classified as moderate (κ = 0.54). When RDT was used as the reference method, sensitivity was 76% and specificity was 83% with an overall agreement classified as moderate-to-substantial (κ = 0.58).

Using expert LM as the main reference method, we found that RMOD detected a higher proportion of *P. vivax* infections than the CareStart® RDT (90% vs. 77%) but less *P. falciparum* infections (78% vs. 92%).

One has to keep in mind, that none of the methods used for comparison is a perfect standard, as they are all subject to systematic and stochastic errors. In fact, we will discuss the data obtained for a considerable set of patients later, where RMOD may have detected low-level infections that were not detected by the other methods.

**Fig. 3:**
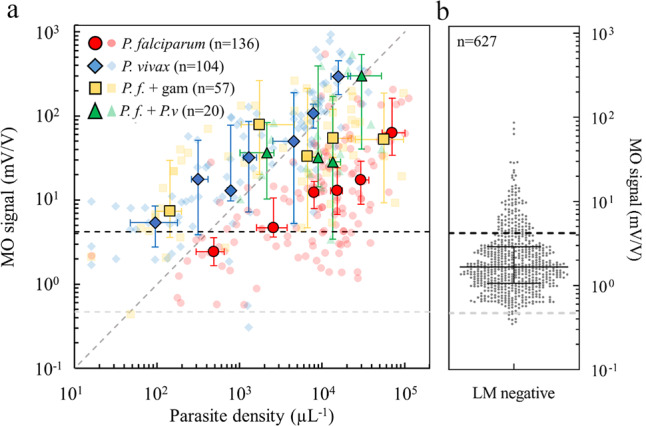
Correlation of MO signals with parasite density as determined by expert LM. **a** MO signals as a function of parasite density for different species, as determined by expert LM. *P. falciparum* mono-infections without gametocytes are represented by red circles, *P. vivax* mono-infections by blue diamonds and *P.f. + P.v*. mixed infections by green triangles. Samples with gametocytes in *P. falciparum* mono-infections (*P.f*. + gam.) are represented by orange squares. The larger, solid-colored symbols show median MO signals and median parasite densities for representative parasite density ranges, with 95% CIs as error bars. The smaller, transparent symbols show the individual test results. The number of biologically independent samples examined over three independent MO experiments is indicated in parentheses. The typical error of the MO signal for a given sample (measured in triplicates) is 11% (individual error bars are not indicated). The diagonal dashed line with a slope equal to 1 represents linear proportionality of the MO signal with parasite density. The dashed horizontal black line represents the cut-off value (MO = 4.19 mV/V) determined by the ROC analysis for all LM positive samples (see Table 2). The dashed horizontal gray line is the median MO background signal (0.47 mV/V) determined in malaria naïve volunteers, which provides a significantly lower cut-off value. **b** MO signals of patient samples with undetectable parasites by expert LM (*n* = 627). Also indicated are the median, 1.67 (95% CI: 1.57–1.78), and the IQR (1.06–2.89) of the MO signals.

Parasite density as determined by LM correlated well with the MO signals as shown in Fig. 3. On average, *P. falciparum*-infected samples resulted in lower MO values as compared to *P. vivax*-infected samples with similar parasite density. *P. falciparum* gametocyte-containing samples formed a characteristic sub-population that, on average, exhibited higher MO signals at a similar parasite density when compared with samples containing only *P. falciparum* asexual stages. The correlation of MO signals with parasite density was better for *P. vivax* (Spearman Rank *R* = 0.83, *p* < 0.0001) than for *P. falciparum* (Spearman Rank *R* = 0.41, *p* < 0.0001).

Despite the clear correlation of MO signal and parasite density as determined by LM, we observed a considerable scatter of the MO signal at a given parasite density. This is natural, as the MO signal depends not only on the density of the parasites but also on the hemozoin content of the parasites, determined by their developmental stage. This alone can explain a considerable variation of the MO signal at any parasite density^26,44^. However, we also observed considerable MO signal scatter within the sample population, which tested negative with all diagnostic methods used for comparison (RDT, LM, and PCR), and patients in this group exhibited, on average, higher MO signals than expected from our previous studies^26^.

**Fig. 4:**
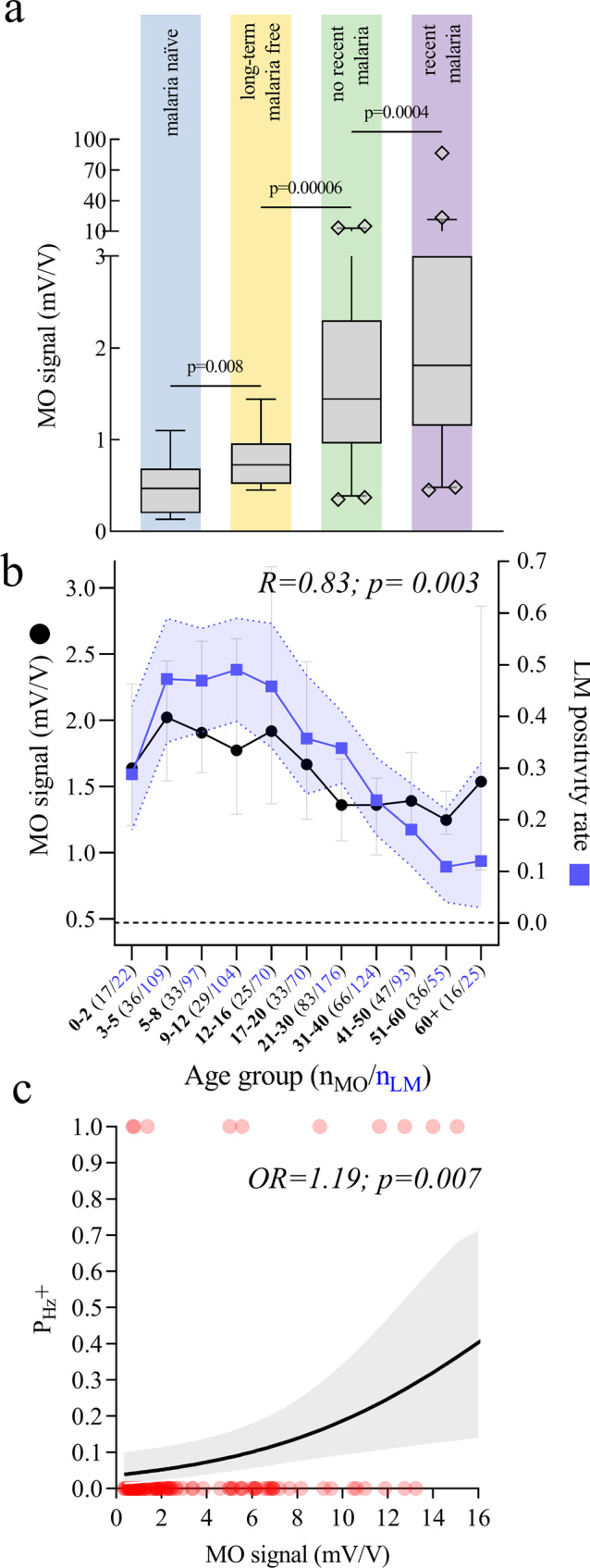
Analysis of MO signals in the patient population found malaria negative by the reference methods. **a** Comparison of MO signals between malaria negative (by LM, PCR and RDT) study participants who reported a recent malaria infection in the two weeks preceding the measurement (*n* = 219) and those who did not report a recent infection (*n* = 294). The graph also shows measurements from *n* = 12 long-term malaria-free volunteers measured during this study, as well as previous data from *n* = 20 malaria naïve volunteers measured in non-endemic settings using the same protocol. All RMOD measurements were done in triplicate. The distributions were compared using two-sided Mann-Whitney tests resulting in the p-values indicated in the graph. The box-and-whisker plots show the median, the IQR, the 1–99 percentile of the MO signals and the individual data points which fall outside the 1–99 percentile range. **b** Correlation between LM-based infection positivity rate and MO signals in patients with undetectable malaria in corresponding age groups. LM positivity rate (blue squares) is shown with the error band representing the 95% confidence intervals (CIs) of proportions. MO signals (black circles) are shown as medians per age group with error bars representing 95% CIs of the medians. The dashed black line corresponds to the median background MO signal in the malaria naïve volunteer population. (0.47 mV/V). The *x*-axis denotes age groups and the number of biologically independent samples (in parentheses, measured in triplicates) per group for MO measurements (*n*_MO_, black) and expert LM (*n*_LM_, blue). **c** Probability of hemozoin-containing leukocyte presence (*P*_Hz+_) versus MO signal in malaria negative samples. The black line and gray-shaded area are the probability prediction and 95% confidence band of the logistic regression model. The red circles are the binary data for hemozoin-containing leukocyte presence (1) or absence (0).

In addition, the median MO signals in the malaria negative population were statistically significantly higher (more than double) than those measured in a smaller sample set of malaria naïve or long-term malaria-free volunteers (*n* = 32), as shown in Fig. 4a.

Furthermore, within the malaria negative study population, patients who indicated having had malaria in the last two weeks exhibited significantly higher MO signals. In summary, this indicates that previous infection is associated with residual hemozoin levels, impacting MO diagnosis of acute malaria.

This hypothesis is further substantiated by the observation that MO signals in the malaria negative (by LM and RDT and PCR) sub-population were highly correlated with malaria positivity rate in the overall population (as determined by LM), when both populations were stratified by age (Fig. 4b, Spearman Rank *R* = 0.83; *p* = 0.003). This implies that either (i) RMOD detects low-level infections that are not detected by the other methods or (ii) there is a higher residual hemozoin level in populations that are more frequently infected with *Plasmodium*.

Evidence that the probability for hemozoin to be present in leukocytes in malaria negative patients is higher for samples with high MO signals is shown in Fig. 4c. Logistic regression indicated that for each unit increase in MO signal, the odds for hemozoin-containing leukocytes to be present in the sample increased by 1.19 (*p* = 0.007).

## Discussion

Improving malaria diagnosis in support of malaria control and elimination, especially through the development of methods applicable in resource-limited settings is important and timely^45^. Malaria diagnosis is complicated by many factors, including the frequent occurrence of asymptomatic infections with low parasite density^46^.

In this study, we tested a magneto-optical method, namely RMOD, for automated and rapid measurement of magnetically induced linear dichroism of hemozoin in close to 1000 human blood samples from suspected malaria patients in a high-transmission area of Papua New Guinea (PNG) where both *P. falciparum* and *P. vivax* are common^47^. In order to evaluate the performance of RMOD, we conducted a comparison with various conventional diagnostic methods, namely expert LM, RDT, and PCR.

RMOD may offer advantages over existing methods in terms of rapidness and cost. While the measurement itself only takes a few seconds, sample preparation and handling are still in the order of minutes. As such, a time-to-result of around 10 min is within reach of the technique. RMOD also has the potential to be cost-competitive. The cost of a prototype as used in the present study is similar to that of a standard light microscope and the cost-per sample is very low as the method is almost reagent-free (see Fig. 1b).

RMOD quantifies the amount of hemozoin in the sample. Correspondingly, we observed a strong correlation between the measured MO signals and parasite density in the peripheral blood samples. This also led to a substantial agreement of RMOD not only with LM but also to moderate-to substantial agreement with RDT and PCR methods^43^. Hemozoin-containing trophozoite and schizont stages are known to sequester less frequently in *P. vivax* than in *P. falciparum* infections, resulting in a higher proportion of these stages present in peripheral blood during *P. vivax* infection^48,49^. Consequently, we observed approximately 10-fold higher average MO signals for *P. vivax* as compared to *P. falciparum* (asexual stages only) at similar parasite densities (compare, e.g., Figs. 2a and 3a). Similarly, for samples containing *P. falciparum* gametocytes, average MO signals were found to be significantly higher in the statistical analyses than for samples containing *P. falciparum* asexual stages only. This is due to the high per-cell hemozoin content found in *P. falciparum* gametocytes^14,22,35^. As a result, RMOD sensitivity and specificity to detect *P. vivax* and mixed infections were significantly higher than that observed for *P. falciparum* when compared to expert LM and PCR. When RDT was used as a reference method, sensitivity and specificity were observed to be higher in pLDH positive samples as these more frequently identify *P. vivax* infections (see Table 2).

As such, our data hint towards RMOD working ‘better’ for *P. vivax*. However, the current results at the same time indicate that RMOD also has a real potential to diagnose *P. falciparum* infections. This is likely due to a combination of reasons: (i) presence (although infrequent) of late *P.f*. stages in peripheral blood^50^; (ii) presence of *P.f*. gametocytes; (iii) the presence of ‘free hemozoin’ released by rupture of sequestered parasites and (iv) the fact that some hemozoin is produced at the ring stage. Our very recent RMOD study, quantifying the hemozoin production during the life cycle of *P. falciparum* parasites shows that ~20% of the hemozoin is produced during ring-stage development^44^.

While MO signals were significantly correlated with parasite density, the scatter of MO signals in the malaria negative population (compare Figs. 3b, 4a) led to considerably higher cut-off levels, below which a measurement is considered negative, than the background MO signal level observed with malaria naïve volunteer samples^25,26^. This led to a seemingly decreased diagnostic performance. The most plausible hypothesis to explain this observation is that a considerable proportion of the patients had hemozoin in their peripheral blood at levels still detectable by RMOD, yet in the absence of an infection detectable by any of the methods used for comparison.

Low-density, asymptomatic infections that are undetectable to expert LM, conventional PCR, and RDTs are highly prevalent in the study area^46^. Such low-level, undetectable infections may still lead to the accumulation of hemozoin in peripheral blood. In addition, hemozoin persists, in the peripheral circulation, usually inside leukocytes^51^. Hemozoin-containing leukocytes can circulate for extended periods after clearance of acute infections^51,52^, which may account, in part, for the high-level background MO signal we observed in the apparently malaria negative population.

*P. falciparum* gametocytes, which, in comparison to asexual stages, contain large amounts of hemozoin, exhibit delayed clearance after antimalarial treatment and may be in circulation for long periods of time, often in very low numbers that are difficult to detect^14,22,53^. Hemozoin is also known to persist in the liver and spleen of previously infected individuals for extended periods of time^54,55^. On this basis, it is likely that in high transmission settings, such as the setting of the present study, elevated hemozoin levels are maintained in the peripheral blood of a large proportion of the general population, either from concurrent low-level infections that are otherwise undetectable^56^, or from previous infections.

First-hand evidence supporting this hypothesis, is shown in Fig. 4. Patients indicating a malaria infection within the previous two weeks exhibited significantly higher MO signals. In addition, samples from *n* = 32 long-term malaria-free or malaria-naïve volunteers exhibited much lower MO signals. When malaria negative patients, who had indicated a recent malaria infection, were excluded from the ROC analyses, overall sensitivity and specificity in comparison to expert LM increased to 86% and 84%, respectively.

It is likely that in low-transmission and/or elimination settings, which many countries are currently working towards, the residual hemozoin level in the population will be lower, and the background MO signal should approach that measured for long-term malaria-free and malaria-naïve volunteer samples. This would lead to an increase in the ability of RMOD to discriminate infections in these settings. In order to estimate the magnitude of improvement, to be expected when lowering the MO signal threshold to the level observed in long-term malaria-free and malaria naïve-individuals, we used the distribution of MO signals obtained from these samples (*n* = 32) together with the distribution of MO signals from samples that were positive for *P. vivax* by expert LM in the present study (*n* = 124) in a ROC analysis. This resulted in an expected sensitivity of 97% and specificity of 97%, together with a near perfect agreement between LM and RMOD techniques, with κ = 0.91. Furthermore, an analogous calculation for the *P. falciparum* samples (*n* = 213) also resulted in 97% sensitivity and 97% specificity with κ = 0.87.

In conclusion, we present an extensive assessment of a promising novel method to diagnose malaria rapidly and at low cost in a high transmission setting and a population of symptomatic, suspected malaria cases in PNG. The present study shows that in such a setting, the RMOD performs well in comparison to expert LM-based diagnosis, the most reliable reference method in our study. However, residual hemozoin levels presented a significant limitation, compromising the ability of RMOD to discriminate between current and previous infections. This limitation is expected to be reduced in low-transmission settings.

In the current state of development, RMOD cannot distinguish between parasite species in *P. falciparum* and *P. vivax* co-endemic settings. Further development of RMOD is ongoing to address species-specific diagnosis, by taking advantage of the species-specific shape and size distributions of hemozoin crystals^57^. It should be noted, that RDTs also cannot clearly distinguish parasite species. For example, the RDT used in this study cannot distinguish mixed species infections^58^, and was able to detect *P. vivax* (based on the pLDH-line test result) with 60% sensitivity and 97% specificity.

While the results of the present study illustrate the potential of RMOD and hemozoin-based malaria diagnosis, they also pose new questions. Future studies are required to further investigate how residual hemozoin levels in different transmission intensity settings, and longitudinal hemozoin dynamics in infected and treated individuals influence RMOD measurement outcomes. It is especially worthwhile considering and further exploring the usefulness of RMOD-based hemozoin detection for malaria diagnosis and in epidemiological studies in low-transmission and elimination settings. The PCR method employed in this study did not achieve an optimal LOD and should thus not be considered as a molecular gold standard. Future studies should include high-volume, ultrasensitive molecular techniques^59,60^ to detect ultra-low density infections and relate their presence to RMOD measurement outcomes.

The current prototype device was deliberately kept flexible so that measurement parameters could be adjusted and optimized. This makes the operation more complex than what we would envision for a finalized diagnostic product, which would focus on ease of use, standardization, and high throughput. For such a final product, we would envision the operation to be very simple at the ‘push of a button’, without requiring any compromise on the present diagnostic performance, which is similar to that of LM.

Many countries in low-transmission or elimination settings experience an increase in the proportion of *P. vivax* infections and it can be expected that *P. vivax* will represent the vast majority of infections in the malaria end-game outside of Africa^61^. Given the rapid and low-cost measurement possible with RMOD and the promising results in particular for *P. vivax*, the method may play a beneficial role in such settings. Since hemozoin is an intrinsic biomarker of *Plasmodium* spp. infection that persists in peripheral blood (similar to the targets of, e.g., serological antibody tests) it may be worth investigating in how far RMOD can be used as a tool to detect transmission hotspots in elimination settings.

## Methods

### Study site and sample collection

Madang is located on the north coast of Papua New Guinea (Fig. 1a). *P. falciparum* and *P. vivax* are highly endemic, and *P. malaria* and *P. ovale* are also present in the study area^38^. Capillary blood samples were collected by qualified nurses or community health workers at Yagaum Rural Hospital located outside of Madang and at Madang Town Clinic in 2017–2019.

Blood samples originated from suspected malaria cases presenting as outpatients at the respective clinics. An RDT (CareStart^®^ Malaria, Accessbio Inc., USA) and two blood slides for LM were prepared at enrollment. All RDT positive patients received standard antimalarial treatment according to PNG malaria treatment guidelines. This study received ethical clearance by the PNG Institute of Medical Research (PNGIMR) Institutional Review Board and the PNG Medical Research Advisory Committee (MRAC, #16.45).

Samples from *n* = 12 long-term malaria-free and *n* = 20 malaria-naïve volunteers were also measured using RMOD and included in the analysis of the present study.

All tests were carried out independently and blinded to the results of each other. Written informed consent was provided by all study participants and, in case of minor patients, from their parents or guardians, in addition to assent from the minor patients.

### Light microscopy

LM was conducted by the PNGIMR microscopy unit consisting of experienced, WHO-certified microscopists. Slides were read, and parasite density determined according to standard WHO guidelines for malaria LM using thick blood smears for parasite density quantification^62^. Blood smears were examined independently by two skilled microscopists, for 200 thick-film fields (×1000 magnification) before being declared *Plasmodium*-negative. Parasite density was calculated from the number of parasites per 200–500 leukocytes (depending on parasite density) and an assumed leukocyte density of 8000 μL^−1^^ 63^. Slides discrepant for positivity/negativity, species determination, or density (>2 log difference) were adjudicated by a WHO-certified level 1 (expert) microscopist. Slides were scored as LM positive for an individual *Plasmodium* species if the species was detected independently by at least two microscopists. Densities were calculated as the geometric mean densities of all positive reads^47^.

### DNA extraction and real-time PCR

DNA extraction was performed on 200 µL of whole blood using DNA extraction kits for 96 well plates (Favorgen Biotech Corp, Taiwan). DNA was eluted in 30 µL of RNAse/DNAse free water. The present study employed a two-step PCR protocol as described previously using molecular probes targeting *Plasmodium 18S rRNA* genes. Briefly, a screening PCR to exclude negative samples was first run on all samples^64^. Samples that were positive by the screening PCR were then subjected to a species-specific quantitative real-time PCR (qPCR) as described elsewhere using a CFX96 Touch qPCR system (Bio-Rad Laboratories Pty., Ltd., Australia)^65^. Therefore, it was possible to quantify parasite density based on gene copy number. For all reactions, 4 µL of the DNA eluate was used, corresponding to DNA material from roughly 26.7 µL of the original blood sample. Primer sequences and thermocycling conditions are provided in Supplementary Information Tables 3 and 4, respectively.

### Rotating magneto-optical detection (RMOD) measurements

The concept of the rotating-crystal magneto-optical setup and the underlying physical principles of hemozoin detection are described in detail in our former studies^24–26^. The working principle is depicted in Fig. 1d. In combination with their magnetism, the elongated, rod or brick-shaped hemozoin crystals exhibit anisotropic optical properties, in particular, magnetically induced linear dichroism^57^. Here, we refer to this effect as the magneto-optical (MO) signal, which is the marker-signal for hemozoin detection and quantification^18,25^.

For the preparation of the blood lysates, 35 µL of whole blood was mixed with 315 µL of lysis solution (13.3 mM of NaOH and 0.03% v/v of Triton X-100 in distilled water) and allowed to stand for at least 10 min to ensure complete lysis. Thereafter, 280 µL of the lysate was transferred into the optical sample holders and measurements were performed without further delay. Samples for RMOD measurements were prepared in triplicate per blood sample.

The hemolysed blood sample, transferred into a cylindrical sample holder, is inserted into the center of a ring-shaped assembly of permanent magnets, which creates a strong uniform magnetic field (**B** ~ 1 T) at the sample position. This magnetic field induces the co-alignment of the hemozoin crystals, and when the magnetic ring is rotated, the co-aligned hemozoin crystals follow this rotation. During the measurement, polarized light from a laser diode is transmitted through the sample in the direction perpendicular to the plane of rotation of the magnetic field. The rotation of the co-aligned dichroic crystals gives rise to a periodic change in the detected intensity. The modulated intensity, divided by the average intensity, corresponds to the measured ‘MO signal’, which is displayed in the corresponding figures in mV/V units^24^.

In general, optical samples were prepared and measured immediately after collection and transport to the laboratory (*n* = 943), but *n* = 22 samples were frozen overnight before preparation and MO measurement due to logistical constraints. The measurement of a single sample took approximately 3–5 min (Fig. 1b).

### Data analysis

Data were entered into an electronic data capture system (Epicollect 5, Imperial College) using standardized data collection forms and into Microsoft Excel 2016 (Microsoft Corporation) spreadsheets. Data analysis and graphing were conducted using Microsoft Excel 2016 (Microsoft Corporation), Graphpad Prism 8.0 (Graph Pad Software), and Stata 13 (StataCorp). No customized computer codes were used.

Proportions are presented alongside their exact 95% confidence intervals^66^. Continuous data are presented as averages (95% confidence intervals) for normally distributed data and medians with interquartile range or range for not normally distributed data.

LM, PCR, and RDT results were used to compare against RMOD results. Following the recommendation of WHO, LM was used as the main reference method. Methods were compared by calculating the sensitivity and specificity, predictive values, and agreement (κ) with respect to the reference method^67^. Receiver operating characteristic (ROC) analyses were used to determine the cut-offs for the continuous RMOD data to best predict presence or absence of infection, i.e., to determine sensitivity and specificity by calculating the minimum distance between the ROC curve and the [0,1] coordinate indicating 100% sensitivity and 100% specificity^68^.

### Reporting summary

Further information on research design is available in the Nature Research Reporting Summary linked to this article.

## Supplementary information

Supplementary Information

Reporting Summary

## Data Availability

All data collected in this study and source data for all tables and figures are provided with this paper as a source data file and also under 10.6084/m9.figshare.13078181.v1 (ref. ^69^). All relevant data are also available from the authors. Source data are provided with this paper.

## Source data

Source Data
