# Supplementary material for: Magneto-optical diagnosis of symptomatic malaria in Papua New Guinea
Source: Nat Commun. 2021 Feb 12;12:969. doi: 10.1038/s41467-021-21110-w (PMC7881035; doi:10.1038/s41467-021-21110-w)
Supplement: Supplementary file 1 — Supplementary Information [file 41467_2021_21110_MOESM1_ESM.pdf]

Supplementary Information: Magneto-optical diagnosis of symptomatic malaria in Papua New Guinea

L. Arndt[1&], T. Koleala[2&], Á. Orbán[3&], C. Ibam[2], E. Lufele[2], L. Timinao[2,4], L. Lorry[2], Á. Butykai[3], P. Kaman[2], A. P. Molnár[3], S. Krohns[5], E. Nate[2], I. Kucsera[6], E. Orosz[6], B. Moore[7], L. J. Robinson[2,8], M. Laman[2], I. Kézsmárki[3,5,#,*], S. Karl[2,4,#,*]

[1] Institute of Natural Materials Technology, University of Technology, Bergstrasse 120, 01069 Dresden, Germany

[2] Vector-borne Diseases Unit, PNG Institute of Medical Research, Madang, P.O. Box 378, Madang Province 511, Papua New Guinea

[3] Department of Physics, Budapest University of Technology and Economics, Budafoki ut 8, 1111 Budapest, Hungary

[4] Australian Institute of Tropical Health and Medicine, James Cook University, 1/14-88 McGregor Road, Smithfield QLD 4870 Australia

[5] Experimental Physics 5, Center for Electronic Correlations and Magnetism, University of Augsburg, Universitätsstr. 1, D-86159 Augsburg, Germany

[6] National Public Health Center, Budapest, Hungary

[7] School of Pharmacy, Curtin University, Kent Street, Bentley, WA, 6102, Australia

[8] Burnet Institute, 85 Commercial Rd, Melbourne VIC 3004, Australia

[&]These authors contributed equally; [#]These authors jointly supervised this work

*Corresponding Authors:

Stephan Karl, stephan.karl@jcu.edu.au;

Istvan Kézsmárki, istvan.kezsmarki@physik.uni-augsburg.de

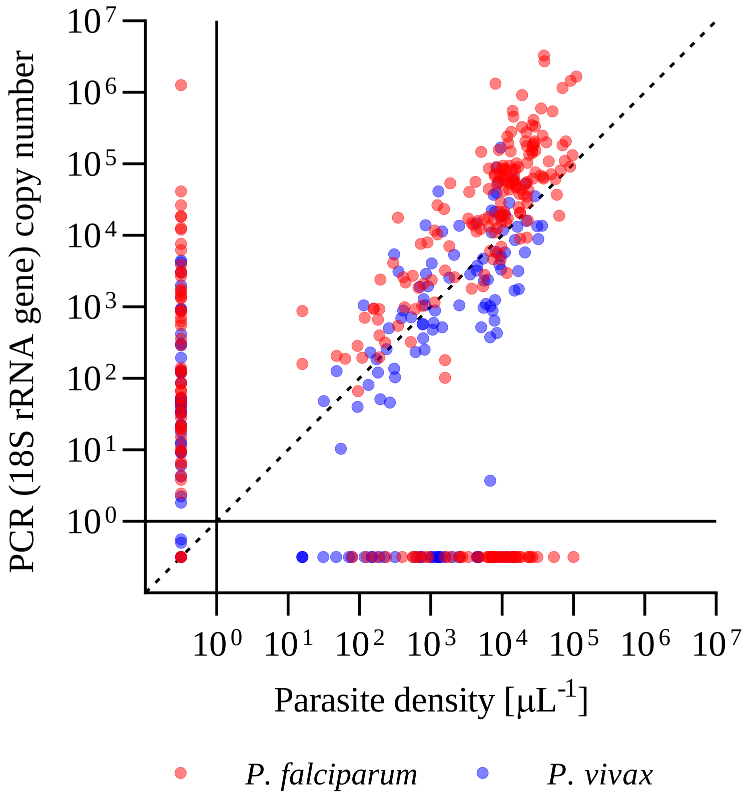

Supplementary Figure 1: Correlation of parasite density, as determined by LM, and PCR (18S rRNA gene) copy number for *P. falciparum* and *P. vivax*. The dashed line represents the line of identity. Symbols displayed at PCR copy <1 and parasite density <1 μL^{-1} represent PCR and LM negative cases, respectively. The effective blood volume subjected to the PCR reaction was 26.7 μL and the estimated limit of detection of the PCR method is on the order of 5 μL^{-1}.

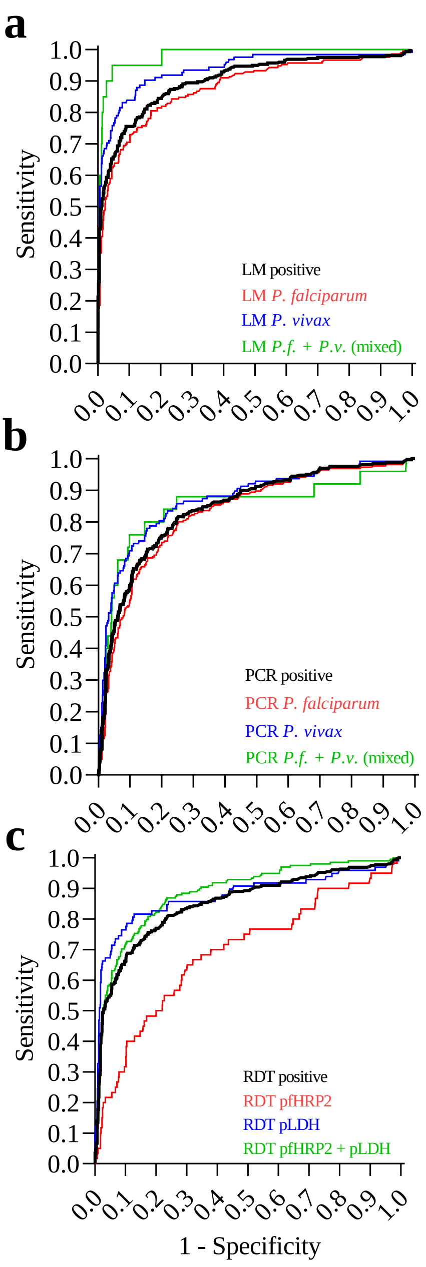

Supplementary Figure 2: Results of the ROC analysis of RMOD versus the conventional diagnostic methods. a) Using expert LM as reference method, the red curve represents *P. falciparum* mono-infections, the blue curve represents *P. vivax* mono-infections and the green curve represents *P.f.+P.v.* (mixed) infections. The black curve represents any positive LM result. **b)** Using PCR as the reference method, the red curve represents *P. falciparum* mono-infections, the blue curve represents *P. vivax* mono-infections, the green curve represents *P.f.+P.v.* (mixed) infections as detected by species-specific qPCR. The black curve is the ROC for RMOD versus any species-specific qPCR result. **c)** Using RDT as the reference method, the red curve represents a positive *PfHRP2* line, the blue curve represents a positive pLDH line, the green curve represents measurements on samples where both lines were positive. The black curve represents any positive RDT result. All panels share a common horizontal scale of 1-Specificity.

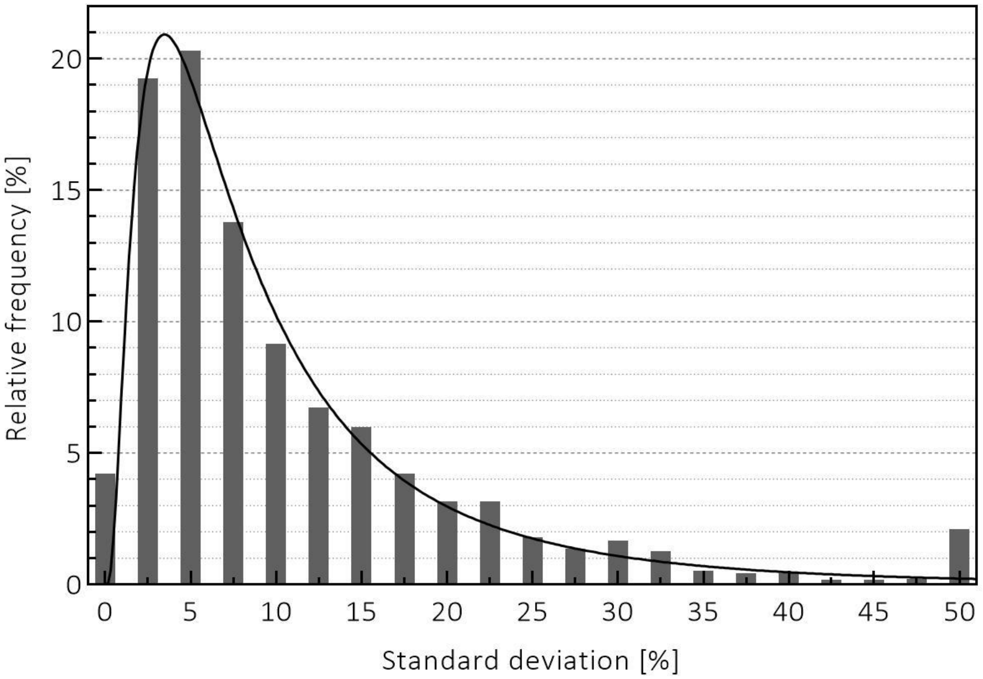

Supplementary Figure 3: The distribution of the standard deviations of the MO signals measured on triplicate blood samples with a log-normal fit. The mean of the standard deviations is 11% (95% CI: 10-12%). Note that the plot has been truncated at x=50% and the n=18 data points (out of N=950) exceeding this value has been merged to the bin-centered at x=50%.

Supplementary Table 1: Diagnostic performance indicators of RDT and PCR methods compared to expert light microscopy as reference standard.

	Sensitivity[a]	Specificity[a]	Positive predictive value[a]	Negative predictive value[a]	κ[a,b]
RDT (any infection)	**87** (82-90)	**88** (85-90)	**78** (74-82)	**93** (91-94)	**0.72** (0.68-0.77)
PCR (any infection)	**80** (75-84)	**89** (86-91)	**78** (74-82)	**90** (88-92)	**0.68** (0.64-0.73)
P.f.-specific PCR[c]	**81** (75-86)	**92** (90-94)	**78** (73-82)	**94** (92-95)	**0.69** (0.64-0.75)
P.v.-specific PCR[c]	**72** (63-80)	**95** (94-97)	**70** (63-77)	**96** (94-97)	**0.67** (0.59-0.74)
mixed infection PCR	**40** (19-64)	**98** (97-99)	**32** (19-49)	**99** (98-99)	**0.34** (0.16-0.52)

[a]Values are given as percentages (bold) and the respective 95% confidence intervals of proportions (in parentheses); [b]κ is the coefficient of agreement according to Cohen, Landis and Koch [1]; [c]includes *P.f./P.v.* detected in mixed infections.

Supplementary Table 2: Cut-off values to characterise sensitivity and specificity resulting from ROC analysis performed using different reference methods (LM, PCR, RDT) and different infection characteristics.

	cut-off	SE (%)[a]	SP (%)[a]	PPV (%)[a]	NPV (%)[a]	AUC[a]	κ[a]
LM result							
all LM positive	4.19	**82** (78-86)	**84** (81-86)	**81** (76-85)	**85** (82-88)	**0.90** (0.88-0.93)	**0.65** (0.59-0.70)
P. falciparum	4.00	**79** (73-84)	**83** (80-86)	**62** (56-67)	**92** (90-94)	**0.88** (0.85-0.91)	**0.57** (0.51-0.63)
P. vivax	5.15	**87** (80-92)	**88** (85-90)	**58** (50-65)	**97** (95-98)	**0.94** (0.91-0.97)	**0.62** (0.55-0.69)
P.f+P.v. (mixed)	10.20	**95** (76-100)	**96** (94-97)	**38** (24-53)	**100** (99-100)	**0.98** (0.96-1.00)	**0.54** (0.40-0.68)
PCR result							
all PCR positive	3.54	**78** (73-82)	**78** (75-81)	**65** (60-70)	**87** (84-90)	**0.84** (0.82-0.87)	**0.54** (0.48-0.5)
P. falciparum	3.01	**80** (74-85)	**75** (71-78)	53 (48-59)	**91** (88-94)	**0.83** (0.80-0.86)	**0.47** (0.41-0.53)
P. vivax	4.84	**79** (71-86)	**84** (81-87)	**50** (43-57)	**95** (93-97)	**0.87** (0.84-0.91)	**0.51** (0.47-0.58)
P.f+P.v (mixed)	5.40	**80** (61-91)	**85** (82-88)	**18** (11-26)	**99** (98-100)	**0.85** (0.75-0.95)	**0.24** (0.15-0.34)
RDT result							
all RDT Positive	4.11	**76** (71-80)	**83** (79-86)	**72** (67-77)	**85** (82-88)	**0.85** (0.82-0.88)	**0.58** (0.52-0.63)
PfHRP2	2.58	**65** (52-77)	**70** (66-73)	**18** (13-26)	**95** (93-97)	**0.69** (0.61-0.77)	**0.16** (0.09-0.22)
pLDH	5.14	**81** (71-88)	**87** (84-90)	**51** (43-59)	**97** (95-98)	**0.87** (0.83-0.92)	**0.55** (0.46-0.63)
PfHRP2+pLDH	4.11	**81** (75-86)	**83** (79-86)	**61** (54-67)	**93** (91-95)	**0.89** (0.86-0.91)	**0.57** (0.51-0.63)

[a]SE, SP, PPV, NPV are the sensitivity, specificity, positive predictive value and negative predictive value, respectively, AUC is the area under the ROC curve, and κ is the coefficient of agreement according to Cohen, Landis and Koch [1]. Bold numbers are the means and numbers in parentheses are the 95% CIs.

Supplementary Table 3. Primer and probe sequences used in the present study, as previously described by Wampfler et al. [2].

A. Generic qPCR (target: conserved regions in 18S rRNA)		
Species	Primer	Sequence (5’ -> 3’)
Plasmodium sp.	QMAL_fw	TTA GAT TGC TTC CTT CAG TRC CTT ATG[a]
	QMAL_rev	TGT TGA GTC AAA TTA AGC CGC AA
	QMAL_probe	FAM-TCA ATT CTT TTA ACT TTC TCG CTT GCG CGA –BHQ
B. Species-specific qPCR		
Species	Primer	Sequence (5’ -> 3’)
P. falciparum (DNA)	Pf_S18S_fw	TAT TGC TTT TGA GAG GTT TTG TTA CTT TG
	Pf_S18S_rev	ACC TCT GAC ATC TGA ATA CGA ATG C
	Pf_S18S_probe	FAM-ACG GGT AGT CAT GAT TGA GTT-MGB-BHQ
P. vivax (DNA)	Pv_18S_fw	GCT TTG TAA TTG GAA TGA TGG GAA T
	Pv_18S_rev	ATG CGC ACA AAG TCG ATA CGA AG
	Pv_18S_probe	HEX-AGC AAC GCT TCT AGC TTA -MGB-BHQ

[a] wobble R = A/G

Supplementary Table 4. Reaction mixtures and PCR profile used in this study, as previously described by Wampfler et al. [2].

<table>
<tr><td colspan="4">A. qPCR Reaction mix[a]</td></tr>
<tr><td rowspan="4">Total volume
14 µL</td><td colspan="3">2X Roche Mastermix (Roche LifeScience, Australia)</td></tr>
<tr><td colspan="3">350nM per primer (forward and reverse)</td></tr>
<tr><td colspan="3">350nM probe (TaqMan, ThermoFisher Scientific, New Zealand)</td></tr>
<tr><td colspan="3">4µl of DNA template</td></tr>
<tr><td colspan="4"></td></tr>
<tr><td colspan="4">B. qPCR Thermocycling profile</td></tr>
<tr><td>Stage</td><td>Step</td><td>Temperature</td><td>Time</td></tr>
<tr><td>Holding</td><td>UDG</td><td>50°C</td><td>2 minutes</td></tr>
<tr><td>Holding</td><td>Activation FastStart Taq DNA Polymerase</td><td>95°C</td><td>15 minutes</td></tr>
<tr><td rowspan="2">Cycling (45x)</td><td>Denature</td><td>95°C</td><td>15 seconds</td></tr>
<tr><td>Anneal/Extend</td><td>60°C</td><td>1 minute</td></tr>
</table>

[a]Reaction mix was prepared on a template-free bench wiped with 2.5M hypochlorite solution. Prepared master mix was added to the reaction plate before transfer to PCR bench for template addition. BioRad 0.2 mL Hard-Shell® 96-Well PCR Plates, low profile, thin wall, skirted, black/white were used for the qPCR.

Supplementary Information References

1 Landis, J. R. & Koch, G. G. The measurement of observer agreement for categorical data. *Biometrics* **33**, 159-174 (1977).

2 Wampfler, R. *et al.* Strategies for detection of *Plasmodium* species gametocytes. *PLoS One* **8**, e76316, doi:10.1371/journal.pone.0076316 (2013).
